# Killer Immunoglobulin-Like Receptor 2DL4 (CD158d) Regulates Human Mast Cells both Positively and Negatively: Possible Roles in Pregnancy and Cancer Metastasis

**DOI:** 10.3390/ijms21030954

**Published:** 2020-01-31

**Authors:** Tatsuki R. Kataoka, Chiyuki Ueshima, Masahiro Hirata, Sachiko Minamiguchi, Hironori Haga

**Affiliations:** Department of Diagnostic Pathology, Kyoto University Hospital, Kyoto 606-8507, Japan; ueshima@kuhp.kyoto-u.ac.jp (C.U.); hiratama@kuhp.kyoto-u.ac.jp (M.H.); minami@kuhp.kyoto-u.ac.jp (S.M.); haga@kuhp.kyoto-u.ac.jp (H.H.)

**Keywords:** allergic reaction, CD158d, FcɛRI, KIR2DL4, KIT, mast cell, pregnancy

## Abstract

Killer immunoglobulin-like receptor (KIR) 2DL4 (CD158d) was previously thought to be a human NK cell-specific protein. Mast cells are involved in allergic reactions via their KIT-mediated and FcɛRI-mediated responses. We recently detected the expression of KIR2DL4 in human cultured mast cells established from peripheral blood of healthy volunteers (PB-mast), in the human mast cell line LAD2, and in human tissue mast cells. Agonistic antibodies against KIR2DL4 negatively regulate the KIT-mediated and FcɛRI-mediated responses of PB-mast and LAD2 cells. In addition, agonistic antibodies and human leukocyte antigen (HLA)-G, a natural ligand for KIR2DL4, induce the secretion of leukemia inhibitory factor and serine proteases from human mast cells, which have been implicated in pregnancy establishment and cancer metastasis. Therefore, KIR2DL4 stimulation with agonistic antibodies and recombinant HLA-G protein may enhance both processes, in addition to suppressing mast-cell-mediated allergic reactions.

## 1. Introduction

Mast cells were first described by Paul Ehrlich in 1878 [1]. Mast cells originate from hematopoietic precursors, and mature in almost all tissues [2,3]. The cells are characterized by their intracellular granules, containing heparin, histamine, serotonin, β-hexosaminidase, prostaglandins (for example in human mast cells, prostaglandin D_2_), growth factors (for example in human mast cells, basic fibroblast growth factor (FGF)/FGF-2, granulocyte macrophage colony-stimulating factor (GM-CSF), nerve growth factor (NGF), vascular endothelial growth factor (VEGF), and stem cell factor (SCF)/KIT ligand/mast cell growth factor), cytokines (for example in human mast cells, tumor necrosis factor (TNF)-α, transforming growth factor (TGF)-β, interleukin (IL)-1β, IL-3, IL-4, IL-5, IL-6, IL-10, IL-11, IL-12, IL-13, IL-16, and interferon (IFN)-γ), chemokines (for example in human mast cells, CC chemokine legend (CCL) 1, CCL2, CCL3, CCL4, CCL5, CCL7, CCL8, CCL11,CCL17, CCL20, CCL22, CXC chemokine legend (CXCL) 2, CXCL8, and CXCL10), and serine proteases (for example in human mast cells, tryptases, chymases, carboxypeptidase A3, granzyme B (GrB), and matrix metalloproteases (MMPs)) [4,5,6]. Mast cells play important roles in both innate and adaptive immune responses by secreting these mediators [4,5,6]. Studies using mast cell-deficient mice, such as Kit^W/Wv^ mice and Kit^Wsh/Wsh^ mice, revealed that mast cells protect against parasitic infections including *Strongyloides ratti* and *Strongyloides brasiliensis* [7,8], as well as the venoms of honeybees or vipers [9]. Mast cells are categorized by the contents of granules. More specifically, human mast cells can be classified into MCT (tryptase-positive and chymase-negative), MCTC (tryptase-positive and chymase-positive), and MCC (tryptase-negative and chymase-positive), while mouse mast cells can be classified into MMC (mucosal type mast cells, which are tryptase-positive and chymase-negative) and CTMC (connective tissue type mast cells, which are tryptase-positive and chymase-positive) [4,5,6]. Mast cells distribute almost all tissues [4,5,6]. MCT or MMC are mainly located in the mucosa of gastrointestinal systems and airways, while MCTC or CTMC are primarily found in the connective tissue like dermis and soft tissues [4,5,6]. Activated gastrointestinal mast cells increase fluid secretion, smooth muscle contraction, peristalsis, and diarrhea. Moreover, activated mast cell in the airways induce airway constriction, increased mucous production, edema, and cough. Activated skin mast cells induce urticaria and angioedema. Thus, mast cells are considered to be as a major effector cell type in allergic diseases including food allergy, asthma, atopic rhinitis, atopic dermatitis, and anaphylaxis [10]. In addition, the roles and functions of mast cells have been focused in autoimmune diseases (Crohn diseases, celiac disease, irritable bowel syndrome, etc.) [11] and cardiovascular diseases (atherosclerosis, etc.) [12]. Mast cell activation and their functions are regulated by cell surface receptors, among which the high-affinity receptor for IgE (FcɛRI) and KIT (CD117/SCF receptor) have been studied extensively [13,14].

FcɛRI expressed on mast cells consists of four subunits: an IgE-binding α chain, a β chain, and two disulfide-bonded γ chains (FcεRIγ) that are the main signal transducers. Among these chains, the β chain plays key roles by amplifying the expression and signaling of FcεRI, and the followed allergic reactions via its immunoreceptor tyrosine-based activation motifs (ITAMs) [15]. When a multivalent antigen-IgE complex binds to FcɛRI on the cell surface, FcɛRI become aggregated or crosslinked, resulting in degranulation and cytokine secretion from the mast cells. KIT is a Type III receptor tyrosine kinase, consisting of an extracellular domain, a juxtamembrane domain, and two tyrosine-kinase domains (TKDs). The TKDs contain a phosphotransferase domain and an ATP binding site. The ligand for KIT, SCF, induces the development, proliferation, maturation, and survival of mast cells. In addition, KIT signaling stimulates cytokine and chemokine release, and augments FcɛRI-mediated responses. The regulation of FcɛRI and KIT should be a promising strategy to control mast cell-mediated allergic reactions [13,14].

Gain-of-function mutations in *KI*T genes, among which D816V is most common, cause the dysregulated cell growth and subsequent clonal accumulation of mast cells in various tissues, a condition referred as mastocytosis [16]. Mastocytosis had been categorized into cutaneous mastocytosis (urticaria pigmentosa) and systemic mastocytosis according to the distribution of neoplastic mast cells, and has been recently recategorized into indolent systemic mastocytosis (ISM), systemic mastocytosis with an associated clonal hematologic non-MC-lineage disease (SM-AHNMD), aggressive systemic mastocytosis (ASM), and mast cell leukemia (MCL) according to the clinical parameters [16]. Patients with mastocytosis often experience mast cell mediator-related symptoms including anaphylaxis, in addition to tissue damage caused by neoplastic mast cell infiltration [17]. To alleviate these symptoms, the numbers of neoplastic mast cells should be reduced in the patients. The regulation on mutated KIT signal pathways should also be a promising approach to control the numbers of neoplastic mast cells. Gain-of-function type KIT mutations are observed in other malignancies, such as gastrointestinal stromal tumor (GIST), seminoma, and acute myelogenous leukemia (AML), though the mutated sites are varied among these malignancies [18].

## 2. Inhibitory Receptors

**Figure 1:**
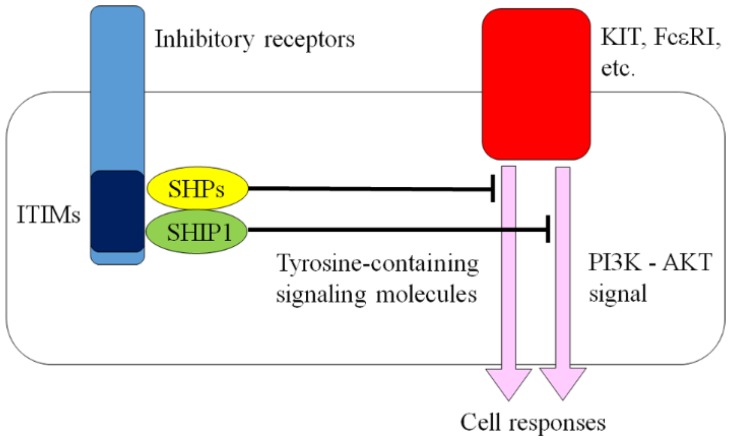
Inhibitory receptor. ITIM: immunoreceptor tyrosine-based inhibitory motif; PI3K: phosphatidylinositol 3-kinase; SHIP: Src homology 2 domain-containing inositol 5-phosphatase; SHP: Src homology 2 domain-containing tyrosine phosphatase.

KIT-mediated and FcɛRI-mediated responses can be modulated by other receptors expressed on the surface of mast cells, including FcγRIIb, Siglecs, mast cell function-associated antigen, signal regulatory protein α, and leukocyte Ig-like receptor B4 (formerly gp49B1), paired Ig-like receptor-B, myeloid-associated immunoglobulin-like receptor I, CD200 receptor, CD300a, CD300f, Allergin-1, 2B4, CD72, programmed death-1 (PD-1), NKp46, carcinoembryonic antigen-related cell adhesion molecule 1, signaling lymphocytic activation molecule family member 8, and killer immunoglobulin-like receptor (KIR) 2DL4 [19,20,21,22,23,24,25,26,27,28,29,30,31]. These include inhibitory receptors characterized by immunoreceptor tyrosine-based inhibitory motifs (ITIMs) within their cytosolic domains (Figure 1) [32]. ITIMs comprise the homology sequence (I/V/L/S)xYxx(L/V) (x; any residue). When the receptors are stimulated, their tyrosine residues become phosphorylated following the activation of receptor or Src family tyrosine kinases. This is followed by the recruitment and activation of non-receptor protein phosphatases, such as Src homology 2 domain-containing tyrosine phosphatase (SHP)-1, SHP-2, and Src homology 2 domain-containing inositol 5-phosphatase (SHIP) 1. SHP-1 and SHP-2 dephosphorylate tyrosine-containing signaling molecules, thus reversing the action of tyrosine kinases. SHIP1 dephosphorylates phosphatidylinositol 3,4,5 trisphosphate at the 3′ position, thereby terminating phosphatidylinositol 3-kinase (PI3K)-driven signaling pathways [32].

Several inhibitory receptors on T and natural killer (NK) cells are classified as immune checkpoint proteins. The discovery of inhibitors against such immune checkpoint proteins, including anti-PD-1 antibodies (nivolumab, pembrolizumab, and cemiplimab), anti-cytotoxic T lymphocyte-associated antigen-4 (CTLA-4) antibody (ipilimumab), anti-lymphocyte activation gene-3 (LAG-3) antibody, anti-T cell immunoglobulin and mucin-domain containing-3 (TIM-3) antibody, anti-T cell immunoglobulin ITIM domain (TIGIT) antibody, anti-V-domain Ig suppressor of T cell activation (VISTA) antibody, and anti-killer immunoglobulin-like receptor (KIR2D) antibody (lirilumab), represents a breakthrough in the field of tumor immunotherapy [33,34]. Anti-PD-L1 (ligand for PD-1) antibody (atezolizumab, avelumab, and durvalumab) and anti-CD200 (ligand for CD200 receptor) antibody (samalitumab) target the ligands for inhibitory receptors on these cells [33,35]. The above-mentioned antibodies interfere with the inhibition of cytotoxic activities of T and NK cells against tumor cells, therefore, they are regarded as “inhibitors” for inhibitory receptors. Gemtuzumab is an antibody against CD33, a member of inhibitory receptors, utilized for the treatment on hematopoietic malignancies [36]. This antibody binds to tumor cells without inducing activation of CD33. To our knowledge, the agonistic antibodies against inhibitory receptors have not been therapeutically utilized.

## 3. KIR2DL4, a Member of the KIR Family

KIRs are human-specific transmembrane proteins which modulate the functions of human NK cells, and some of them are members of inhibitory receptors [37]. NK cells can kill major histocompatibility (MHC) class I-negative tumor cells but cannot kill MHC class I-positive tumor cells. To explain this observation, the missing self-hypothesis had been proposed. This hypothesis predicted that NK cells express MHC class I-receptors, transducing inhibitory signals. KIRs had been identified as such receptors (at the time, KIRs termed killer “inhibitory” receptors). Then, it revealed that the at least 14 KIR genes have been identified in the human genome and are clustered on chromosome 19q13.4 (then, KIRs are called killer “immunoglobulin-like” receptors) [37]. The number and combination of KIR genes in the genome varies within the human population. Additionally, the expression of each KIR is regulated at the transcriptional level by DNA methylation. Therefore, KIR protein expression repertoires are varied within the human population. The KIR nomenclature reflects the structure of the proteins: the first two characters correspond to the number of the extracellular domains (2D and 3D), and the third digit corresponds to the length of the cytoplasmic tail (L or S). KIRs with a long cytoplasmic domain (L) contain ITIMs (KIR-L), while those with a short cytoplasmic domain (S) lack ITIMs (KIR-S) [37]. Therefore, KIR-Ls are categorized into the inhibitory receptors, and expected to transduce inhibitory signals to NK cells.

Unlike other KIR members, KIR2DL4 is constitutively expressed in all NK cells on the transcriptional level [38]. Human leukocyte antigen (HLA)-G has been identified as the ligand for KIR2DL4 [38,39]. HLA-G is a non-classical HLA class I molecule, and composed of four membrane-bound (HLA-G1, -G2, -G3, and -G4) and three soluble (HLA-G5, -G6, and -G7) isoforms [39]. These isoforms are generated by alternative splicing of HLA-G mRNA. In addition to KIR2DL4, CD85j/immunoglobulin-like transcript 2 (ILT2), CD85d/ILT4, CD8, and CD160 have also been reported to bind HLA-G [39]. CD85j is expressed by monocytes, B cells, dendritic cells (DCs), myeloid derived suppressive cells (MDSCs), NK cells, and T cells. CD85d is expressed by DCs, monocytes, neutrophils, and MDSCs. KIR2DL4 contains two extracellular domains and a long cytoplasmic domain, therefore has been classified as a KIR-L [37]. The ITIM of KIR2DL4 protein has been shown to interact with SHP-1 and SHP-2, like other inhibitory receptors, and the followed inhibition of CD16/FcγRIIIa signaling in human NK cells [40]. CD16 mediates antibody-dependent cell-mediated cytotoxicity, therefore KIR2DL4-mediated CD16 inhibition is in line with the missing self-hypothesis. In contrast to such inhibitory activity, KIR2DL4 stimulation induces weak cytotoxicity and the secretions of IFN-γ, TNF-α, IL-1α, IL-1β, IL-6, and IL-8 from human NK cells, mediated by activating signals via FcεRIγ independent of the presence of ITIM [41,42,43,44]. These responses could potentially enhance tumor and virus elimination, although their physiological roles have not been established. Soluble HLA-G has been shown to induce similar cytokine secretions [44]. It is thought that soluble HLA-G binds to KIR2DL4 in endosomes and activates DNA-PKcs (DNA-dependent protein kinase, catalytic subunit)–AKT–NF-κB signals [44,45]. The expression and function of KIR2DL4 in other immune cells remains poorly understood.

## 4. KIR2DL4 Expression in Human Mast Cells

Similar to NK cells, mast cells secrete the Th1 cytokine IFN-γ [46,47] and show cytotoxic activity by producing GrB [48,49]. Therefore, we hypothesized and explored the expression of KIR2DL4 in human mast cells.

We detected the expression of KIR2DL4 in human cultured mast cells established from the peripheral blood of healthy volunteers (PB-mast) [50], in a human mast cell line LAD2 expressing normal KIT and normal FcεRI [51], and in human tissue mast cells [30]. We could not detect the expression of other HLA-G receptors, such as CD85j, CD85d, CD8, and CD160, in these cultured mast cells. In contrast, we observed that the KIR2DL4 protein expression was lacking in the human neoplastic mast cell line HMC1.2 expressing mutated KIT and deficient in FcεRI expression [52], and that nine of 15 cutaneous mastocytosis samples were KIR2DL4-negative [30]. These observations suggest that a lack of KIR2DL4 protein expression could serve as a diagnostic marker of neoplastic changes in mast cells, as is the case that lack of or decreased expression of KIR2DL4 is detected in neoplastic NK cells, NK cell lymphoma [53,54].

## 5. Possible Regulation by KIR2DL4 Stimulation on Mast Cell-Associated Allergic Reactions and Mastocytosis

Both PB-mast and LAD2 cells have been used to examine the role of KIR2DL4 in FcɛRI-mediated and/or KIT-mediated reactions of human mast cells. Treatment of PB-mast and LAD2 cells with two agonistic antibodies against KIR2DL4 suppressed FcɛRI-mediated degranulation and KIT-mediated growth of these cells [30]. These results suggested that KIR2DL4 stimulation is expected to suppress mast cell-mediated allergic reactions. In addition, administration of the same antibodies induced the secretion of the serine protease, GrB [30]. The inhibitory effects on FcɛRI-mediated and KIT-mediated responses, as well as the GrB secretion, were abrogated when a SHP-2 inhibitor was used [30], suggesting that the KIR2DL4-mediated responses were SHP-2-dependent [30]. SHP-2 regulates cell functions both positively and negatively; SHP-2 enhances cell functions by activating Grb2-associated binder family member (Gab) 2–mitogen-activated protein kinase (MAPK) signal pathways, and suppresses cell functions by dephosphorylating phopho-proteins that are involved in various other signal pathways [55]. In human mast cells, KIR2DL4-induced suppression of FcɛRI-mediated and KIT-mediated responses would be mediated by the dephosphorylating activity of SHP-2, and at the same time KIR2DL4-induced GrB secretion would be mediated by the Gab2–MAPK signal pathway. We observed that KIR2DL4-induced GrB secretion was c-Jun N-terminal kinase (JNK)-dependent in human mast cells [30], therefore KIR2DL4–SHP-2–Gab2–JNK signaling would exist in human mast cells.

As mentioned above, interference with the KIT-mediated and FcεRI-mediated signal pathways has been proposed as a potential strategy to control mast cell-mediated allergic reactions [13,14], highlighting the importance of KIR2DL4 as a target for allergic diseases. In other words, the agonistic antibodies against KIR2DL4 would be useful for allergy therapy, similar to the anti-IgE antibody omalizumab clinically utilized to neutralize IgE in blood, and eliminate of FcɛRI-mediated function and control mast cell-mediated allergic reactions [56]. Imatinib is known to inhibit KIT signal pathways, and the efficacy of this drug is shown in patients with severe refractory asthma, one of the mast cell-mediated allergic diseases [57]. KIR2DL4 can inhibit both KIT-mediated and FcεRI-mediated signal pathways, therefore the agonistic antibodies against KIR2DL4 could potentially exert synergistic effects in combination with omalizumab and imatinib.

Some human mastocytosis (six of 15 cutaneous mastocytosis cases) expressed KIR2DL4 protein, however, whether KIR2DL4 stimulation suppresses the growth of KIT-mutated neoplastic mast cells in vitro has not been determined. It is believed that KIR2DL4 may inhibit the growth of KIT-mutated neoplastic mast cells. This hypothesis is supported by the fact that PD-1-mediated SHP-2 activation could inhibit the growth of KIT-mutated neoplastic mast cells [26]. Moreover, avapritinib is a recently identified KIT D816V inhibitor and shown to be useful for mastocytosis treatment [58]. Another KIT inhibitor imatinib is ineffective on KIT D816V observing almost all mastocytosis [59] but is effective on KIT mutations in GISTs [60]. Resistance to this drug, mainly caused by the second mutation in KIT gene, is a problem arising during imatinib therapy on GISTs [60]. KIR2DL4-targeted therapy might be useful, especially when a second mutation in the *KIT* gene is caused during avapritinib-utilized mastocytosis therapy.

## 6. Involvement of KIR2DL4 on Human Mast Cells in the Establishment of Pregnancy

The natural ligand of KIR2DL4 is HLA-G, as mentioned above [38,39]. The HLA-G expression was physiologically restricted in trophoblasts, cornea, thymic medulla, and islets of pancreas [39]. HLA-G is involved in tumor progression, viral infection, organ transplantation, autoimmune and inflammatory diseases [39]. Furthermore, soluble HLA-G levels have been associated with allergen-specific IgE levels in the serum of patients with allergic rhinitis [61]. Herein, we then focused on the interaction of human mast cells expressing KIR2DL4 with HLA-G-positive trophoblasts during pregnancy establishment and with HLA-G-positive cancer cells during cancer progression.

Interactions between KIR2DL4 and HLA-G have been investigated in the context of decidual NK cell-trophoblast interactions during the establishment of pregnancy [62]. The reduced expression of KIR2DL4 protein in decidual NK cells was observed in some women with recurrent spontaneous abortion [63]. KIR2DL4 is expressed on human decidual NK cells, and suppresses the cytotoxic activity against the HLA-G-expressing fetuses [62,63]. Therefore, the reduced KIR2DL4 expression levels on decidual NK cells have been thought to increase the susceptibility of NK cell-mediated cytotoxic activity and the following recurrent spontaneous abortion [63]. Regulatory T cells (Tregs) have also been also implicated in the establishment of pregnancy [64]. Reduced numbers of decidual Tregs were observed in some women with recurrent spontaneous abortion [65,66,67]. Decidual Tregs is necessary for the tolerance toward semi-allogenic fetuses [65,66,67]. Thus, the studies on the roles of decidual immune cells have been focused on the suppression of semi-allogenic fetus rejections in the establishment of pregnancy. Additionally, recent studies show that decidual immune cells are necessary for angiogenesis in the establishment of pregnancy [68]. For example, decidual NK cells secrete angiogenic factors, such as VEGF, angiopoietin-2, placental growth factor (PlGF), and chymase [69,70]. Decidual NK cells are thought to secrete these factors, induce angiogenesis and spiral artery remodeling. Recently, a new subset of decidual NK cells, pregnancy trained decidual NK cells (PTdNKs) has been characterized as an enhancer of proper placentation, which increases the secretion of VEGF which supporting angiogenesis [71].

Another immunocompetent cell, mast cell, is also distributed to the uterus [72]. Mast cells are identified in the endometrium throughout the menstrual cycle, and the activation of mast cells are observed prior to menstruation [72]. Nevertheless, mast cells had been thought to be indispensable for pregnancy; mast-cell-deficient Kit^W/Wv^ and Kit^Wsh/Wsh^ mice are infertile, though blastocyst transfer can archive implantation and live births in both mice [73,74]. Moreover, mast cell transfer to uterus could improve the success ratio of establishment of pregnancy in Kit^Wsh/Wsh^ mice [75]. Mast cell chymase was subsequently shown to be important for angiogenesis in the decidual tissues of mice and humans, as is the case of NK cell-derived chymase [65]. In addition, mast cells produce other angiogenic molecules such as VEGF, bFGF, heparin, histamine, SCF, as mentioned above [4,5,6]. Transfer of Tregs into abortion-prone mice promoted the expansion of uterine mast cells and the angiogenesis, resulting in the improvement of the success ratio of establishment of pregnancy [76].

We observed that mast cells in the decidual tissues of parous women expressed KIR2DL4 [31]. In contrast, the numbers of decidual mast cells and KIR2DL4 expression was significantly reduced in infertile women long-term treated with corticosteroids for autoimmune diseases, liver transplantation, or kidney transplantation [31]. The numbers of NK cells and Tregs in decidual tissues were not significantly different among the infertile women long-term treated with corticosteroids, infertile women of uncertain etiology, and the parous women, as is not the case of mouse experiments. We suspected that KIR2DL4 on decidual mast cells seemed to be involved in the establishment of pregnancy. To elucidate the importance of the interaction between KIR2DL4 on mast cells and HLA-G on trophoblasts, we co-cultured a HLA-G-positive human trophoblast cell line HTR-8/SVneo cells [77] with a human mast cell LAD2. The co-culture showed enhanced migration and tube formation of HTR-8/SVneo in the KIR2DL4-HLA-G interaction-dependent manner [31]. When KIR2DL4 was stimulated, LAD2 cells secreted leukemia growth factor (LIF) and a serine protease MMP-9 [31]. LIF is a member of the IL-6 family of cytokines [78]. LIF receptor consists of gp130 and LIF receptor β subunit, and transduces the Janus kinase (JAK)–signal transducer and activator of transcription (STAT) signaling pathway [78]. LIF-knockout female mice are infertile due to embryo implantation failure [78]. LIF is highly expressed in the endometrial glands, as well as decidual NK and mast cells [31,78]. LIF enhances the invasion and differentiation of trophoblasts, resulting in the implantation of fetuses [78]. LIF also enhances tumor progression by promoting cell cycle progression and invasive activity of tumor cells via STAT3 activation, as is the case of other IL-6 family of cytokines [79]. Similarly, KIR2DL4-induced LIF secretion by LAD2 enhanced the migration of HTR-8/SVneo via STAT3 activation. Serine proteases, including MMP-9, induce the degradation of protease-activated receptors [80], which subsequently decreases in the secretion of soluble fms-like tyrosine kinase-1 (sFlt-1), an inhibitor of VEGF, from trophoblasts [81]. KIR2DL4-induced MMP-9 secretion from LAD2 decreased the secretion of sFlt-1 from HTR-8/SVneo, and the followed increase of tube formation by HTR-8/SVneo. Thus, mast cell deficiency in decidual tissues leads to pregnancy and parturition disorder, and KIR2DL4 downregulation is associated with infertility, suggesting that selective KIR2DL4-induced production of LIF and MMP-9 by mast cells that may illustrate the critical context-specific role of mast cells in pregnancy.

## 7. Involvement of KIR2DL4 on Human Mast Cells in Tumor Progression

HLA-G is expressed in various tumors, as mentioned above [38,39]. The expression of HLA-G in neoplasms was first identified in choriocarcinomas, neoplastic trophoblastic cells [82], and secondly identified in malignant melanoma [83]. HLA-G expression has also been reported in lung cancer, oral and nasopharyngeal squamous cell carcinoma, esophageal cancer, gastric cancer, colorectal cancer, hepatocellular carcinoma, pancreatic cancer, uterine cancer (cervical cancer and endometrial cancer), ovarian cancer, glioblastoma, malignant lymphoma, and so on. Additionally, HLA-G expression levels have been associated with advanced tumor stage, metastasis status and poor diagnosis in various tumors [84]. This is partially explained by the fact that HLA-G is thought to suppress the cytotoxic activity of human NK cells against HLA-G-positive tumor cells via KIR2DL4 or other receptors, such as CD85j, CD85d, CD8, and CD160 [84]. Therefore, HLA-G could be a target for immune checkpoint therapy, though the HLA-G-targeted drugs have not been therapeutically utilized to our knowledge. Breast cancer cells also express HLA-G, and HLA-G expression in breast cancers is associated with poor prognosis [85,86,87,88].

A role for mast cells in tumor progression has been under discussion [89,90]. When mast cells were first described by Paul Ehrlich, he reported mast cells distributed around skin cancers and pointed out the association between mast cell and tumorigenesis [1]. Experimental tumorigenesis after subcutaneous treatment with 3-methylcholanthrene revealed that the tumor incidence in mast cell-deficient KIT^W/Wv^ mice was increased compared to that in control mice, therefore mast cells had been thought to be involved in tumor suppression [91]. This finding could be attributed to the fact that mast cells produce anti-tumor mediators, such as granzyme B, reactive oxygen species, and Th1 cytokines, including TNF-α and IFN-γ [46,47,48,49]. Mast-cell-produced IL-9 were shown to inhibit tumor cell engraftment [92]. Mast cells are a major source of histamine, and histamine was shown to inhibit tumor growth by promoting the development of monocyte-derived DCs [93]. The combined deficiency in mast cell chymase, tryptase, and carboxypeptidase A3 was associated with reduced invariant NKT cells and increased melanoma dissemination [94]. Contrary to these reports on suppressive roles of mast cells in tumor progression, there are reports on enhancing roles of mast cells. The association between mast cells and tumor angiogenesis has been focused in this area. Mast cells produce angiogenetic mediators, such as VEGF, bFGF, heparin, histamine, SCF, IL-8, NGF, TNF-α, tryptase, in forming tumor vessels and the followed invasion or metastasis of cancers [87,88,93]. Mast cells produce Th2 cytokines which contributes to M2 (pro-tumor) polarization of tumor-associated macrophages, and the cells produce TNF-α and IL-10 which promote the Treg-mediated immune tolerance and immune tolerance against tumors [4,5,6]. Mast cells produce TGF-β, CXCL8, and TNF-α, promoting epithelial-to-mesenchymal transition in tumor invasion and metastasis [94,95,96]. Mast-cell-produced histamine was shown to inhibit hypoxia inducible factor-1 alpha expression and the followed growth suppression in melanoma [97]. Mast cells producing serine proteases such as tryptase and MMPs [4,5,6] degrade the extracellular matrix to increase the angiogenesis, resulting in metastasis [98]. The association between infiltrating mast cells and tumor angiogenesis has clinically been shown in pulmonary carcinoma, gastric carcinoma, colorectal carcinoma, endometrial carcinoma, cervix carcinoma, prostatic carcinoma, skin tumors including basal cell carcinoma and melanomas, lymphomas, multiple myeloma, myelodysplastic syndrome, and leukemia [89,90]. Thus, mast cells play dual roles in tumor progression, and the classification into anti-tumorigenic MC1 and pro-tumorigenic MC2 mast cell types have been advocated recently [90]. In breast cancer cells, mast cell infiltration is also related to increased angiogenesis and poor prognosis [99,100]. We examined the association between HLA-G, its receptor KIR2DL4, mast cells, and breast cancer progression.

Using clinical samples, we have shown that HLA-G-positive breast cancer cells interact directly with KIR2DL4-positive tissue mast cells immunohistochemically [30]. The interaction is associated with lymph node metastasis and lymphovascular invasion [30]. Thus, KIR2DL4 on mast cells seems to be involved in cancer progression. To elucidate the importance of the interaction between KIR2DL4 on mast cells and HLA-G on cancer cells, we co-cultured the HLA-G-positive human breast cancer cell line MCF-7 cells [101] with the human mast cell LAD2. The co-culture showed enhanced invasion of MCF-7 in a KIR2DL4–HLA-G interaction-dependent manner [30]. MMP-9 secreted from KIR2DL4-stimulated LAD2 cells were found to be involved in this process [30]. Thus, human mast cells are associated with an invasive phenotype of HLA-G-positive breast cancers.

## 8. KIR2DL4 as a Potent Therapeutic Target

KIR2DL4 can be activated by recombinant HLA-G or by agonistic antibodies, such as clone 181,703 and clone 33. The ability of these molecules to enhance the establishment of pregnancy suggests their therapeutic use in the treatment of infertility, in addition to allergic diseases and mastocytosis. KIR2DL4 is expressed by human NK cells, and KIR2DL4-targeted drugs are expected to enhance NK activity and to induce IFN-γ secretion [46,47,48,49]. Therefore, KIR2DL4-targeted drugs might enhance NK activity and the following enhancing defensive effects against virus infections. However, KIR2DL4-targeted drugs might enhance HLA-G-positive cancer progression, and patients treated with KIR2DL4 stimulants should first be carefully screened for the presence of malignancy. Additionally, KIR2DL4 expression has been detected in dendritic cells [102]. KIR2DL4-targeting therapies may exert undesirable effects by modulating the function of these cells.

The IL-33/ST2 signal pathway and Mas-related G protein-coupled receptor X2 also play important roles in mast cell biology [14], and the effects of KIR2DL4 on the function of these receptors should be elucidated before KIR2DL4-targeting therapies can enter into clinical practice.

## 9. Conclusions

**Figure 2:**
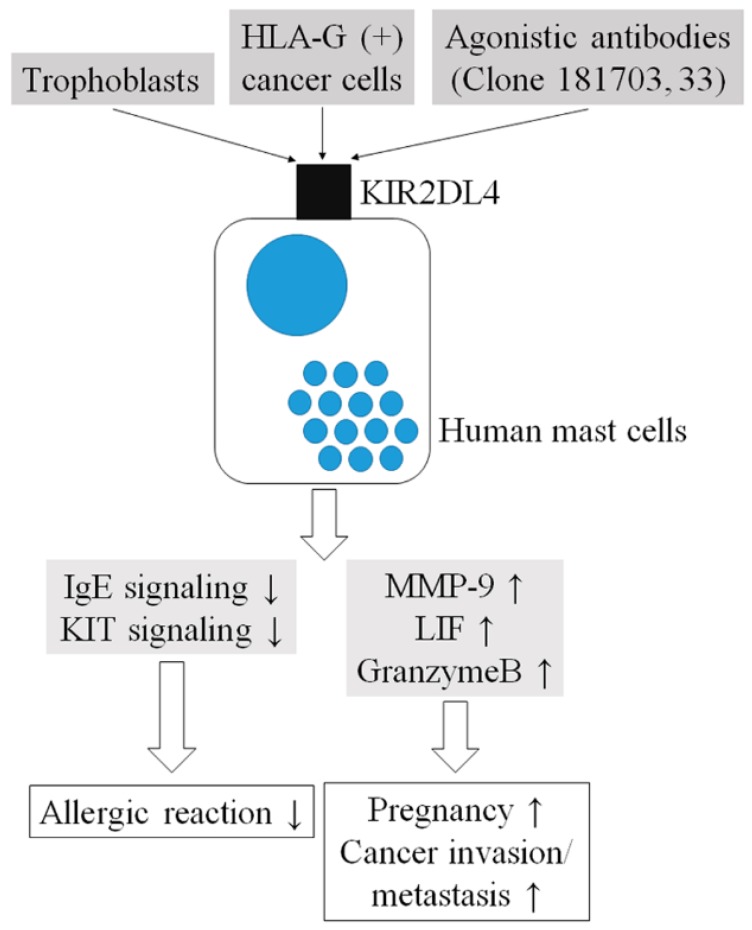
The current model. HLA: human leukocyte antigen; KIR: killer immunoglobulin-like receptor; LIF: Leukemia inhibitory factor; MMP: matrix metalloprotease.

KIR2DL4, a member of the KIR family, is expressed by human mast cells. It positively and negatively regulates the functions of human mast cells such that its stimulation may suppress mast cell-mediated allergic reactions and enhance the establishment of pregnancy (Figure 2).

## Abbreviations

AML	acute myelogenous leukemia
ASM	aggressive systemic mastocytosis
CTLA-4	cytotoxic T lymphocyte-associated antigen-4
DC	dendritic cell
DNA-PKcs	DNA-dependent protein kinase, catalytic subunit
FcεRIγ	Fc receptor γ chain
FGF	fibroblast growth factor
Gab	Grb2-associated binder family member (Gab)
GIST	gastrointestinal stromal tumor
GM-CSF	granulocyte macrophage colony-stimulating factor
GrB	granzyme B
HLA	human leukocyte antigen
IFN	interferon
IL	interleukin
ILT	immunoglobulin-like transcript
ISM	indolent systemic mastocytosis
ITAM	immunoreceptor tyrosine-based activation motifs
ITIM	immunoreceptor tyrosine-based inhibitory motif
JAK	Janus kinase
JNK	c-Jun N-terminal kinase
KIR	killer immunoglobulin-like receptor
LAG-3	lymphocyte activation gene-3
LIF	leukemia growth factor
MAPK	mitogen-activated protein kinase
MCL	mast cell leukemia
MDSC	myeloid derived suppressive cell
MHC	major histocompatibility
MMP	matrix metalloprotease
NK	natural killer
NGF	nerve growth factor
PB-mast	human cultured mast cells established from peripheral blood derived from healthy volunteers
PD	programmed death
PI3K	phosphatidylinositol 3-kinase
PlGF	placental growth factor
PTdNK	pregnancy trained decidual NK cell
SCF	stem cell factor
sFlt-1	soluble fms-like tyrosine kinase-1
SHIP	Src homology 2 domain-containing inositol 5-phosphatase
SHP	Src homology 2 domain-containing tyrosine phosphatase
SM-AHNMD	systemic mastocytosis with an associated clonal hematologic non-MC-lineage disease
STAT	signal transducer and activator of transcription
TGF	transforming growth factor
TIGIT	T cell immunoglobulin ITIM domain
TIM-3	T cell immunoglobulin and mucin-domain containing-3
TKD	tyrosine-kinase domain
TNF	tumor necrosis factor
Treg	regulatory T cell
VEGF	vascular endothelial growth factor
VISTA	V-domain Ig suppressor of T cell activation
